# Mental health literacy of adolescents in Bermuda, according to age, gender and race

**DOI:** 10.1093/heapro/daae131

**Published:** 2024-10-14

**Authors:** Daniel Cavanagh, Anthony Jorm, Nicola Reavley, Shawnee Basden, Laura M Hart

**Affiliations:** Centre for Mental Health and Community Wellbeing, Melbourne School of Population and Global Health, University of Melbourne, 207 Bouverie St, Carlton VIC 3053, Australia; Centre for Mental Health and Community Wellbeing, Melbourne School of Population and Global Health, University of Melbourne, 207 Bouverie St, Carlton VIC 3053, Australia; Centre for Mental Health and Community Wellbeing, Melbourne School of Population and Global Health, University of Melbourne, 207 Bouverie St, Carlton VIC 3053, Australia; Department of Arts and Science, Bermuda College 21 Stonington Avenue, Paget PG 04, Bermuda; Centre for Mental Health and Community Wellbeing, Melbourne School of Population and Global Health, University of Melbourne, 207 Bouverie St, Carlton VIC 3053, Australia

**Keywords:** adolescent, mental health literacy, problem recognition, Caribbean, depression, social anxiety

## Abstract

Mental health literacy (MHL) is an important part of the help-seeking process, yet there is a lack of knowledge about the MHL of adolescents in the Caribbean. This region is important to study as it is underrepresented in mental health research globally. The aim of this study is to explore the ability of adolescents in Bermuda to recognize depression and social phobia (social anxiety) and their beliefs about the sources of help for a peer with these mental health problems. This cross-sectional study surveyed middle and high school students aged 10–19 years in Bermuda. Online surveys conducted between November 2022 and June 2023 gathered demographic data including age, gender and race, and assessed the ability to recognize depression and social anxiety from descriptions provided in randomly assigned vignettes, and beliefs about sources of help. Across 15 middle and high schools, 2423 adolescents (out of 3593 eligible participants) completed all demographic and MHL survey questions (1139 males, 1272 females). Recognition rates for depression and social anxiety were 60% and 53%, respectively. Compared to females, males endorsed a greater variety of help sources. Reporting symptoms of depression or anxiety reduced the likelihood of endorsing multiple sources of help. MHL of adolescents in Bermuda is sub-optimal, particularly for social anxiety. Mental health promotion programs may be useful in improving recognition rates.

**Contribution to Health Promotion**

- New insights are provided about the mental health literacy of adolescents in the Caribbean, a region underrepresented in research.
- More than two out of five adolescents could not recognize depression or social anxiety from a vignette.
- A small minority considered health professionals as harmful to a peer with a mental health problem.
- Health promotion for both adolescents and adults working with adolescents is necessary to improve community knowledge and attitudes.

## BACKGROUND

Mental health literacy (MHL) refers to the ‘knowledge and beliefs about mental disorders which aid their recognition, management or prevention’ (Jorm *et al*., 1997). MHL is important as it has been found that when individuals have better knowledge about mental disorders they are more likely to seek appropriate help (Bonabi *et al*., 2016; Gorczynski *et al*., 2017; Stolzenburg *et al*., 2019). Adolescents in developed countries are known to have lower rates of professional help-seeking compared to adults for their mental health problems (Burns and Rapee, 2006; Reavley and Jorm, 2011b; Marcus *et al*., 2012; Yap *et al*., 2012). Low rates of help-seeking are a concern as adolescence is the stage of life where mental disorders often have first onset (Kessler *et al*., 2007), and delays in help-seeking can lead to greater severity and impairment (Wang *et al*., 2007) and a higher risk of developing comorbidity (Altamura *et al*., 2014, 2022). Improving population mental health outcomes requires both health service reform and actions to improve community attitudes to mental health and help-seeking. MHL research is vital to inform the actions and policies developed to improve community attitudes that are most needed for each life stage, such as improving adolescents’ knowledge of mental disorder symptoms and mental healthcare services.

The ability to correctly recognize mental disorders (problem recognition) is a fundamental component of MHL as it is considered a necessary first step in seeking help from a professional (Wright *et al*., 2007; Thompson *et al*., 2008), and failure to recognize symptoms can lead to a delay in help-seeking (Reavley and Jorm, 2011b). Recognition rates tend to be higher in developed countries (Reavley and Jorm, 2011b; Loureiro *et al*., 2013), and, with effective mental health promotion, have been shown to improve over time (Reavley and Jorm, 2012). However, many studies of school-aged adolescents indicate that > 50% correctly recognize depression (Melas *et al*., 2013; Skre *et al*., 2013; Adeosun, 2016; Coles *et al*., 2016; Ogorchukwu *et al*., 2016; Ahmad *et al*., 2022; Singh *et al*., 2022), with even lower recognition for social phobia (hereafter referred to as social anxiety) (Olsson and Kennedy, 2010; Arslan and Karabey, 2023). Another important component of MHL relates to help-seeking beliefs, including beliefs about the helpfulness of sources of support for a peer with an MHP. Studies on help-seeking tend to focus on personal help-seeking intentions finding that adolescents tend to prefer getting initial help for mental health problems from family and friends (Wright *et al*., 2005; Jorm and Wright, 2007; Yap *et al*., 2012). As such, it is important to understand who the friends of peers with mental health problems suggest as further sources of support. For depression and social anxiety, adolescents in developed countries tend to suggest lay help-seeking such as family and friends rather than professional sources of help such as counsellor, general practitioner or psychiatrist (Gonzalez *et al*., 2005; Wright *et al*., 2005; Coles *et al*., 2016), although mental health professionals such as psychologists have also received high endorsement in countries including India, Ireland and Australia (Byrne *et al*., 2015; Ogorchukwu *et al*., 2016; Lubman *et al*., 2017; Sharma *et al*., 2017).

Effective mental health promotion planning is limited in regions such as the Caribbean by a lack of locally collected data. Relevant research typically explores health literacy rather than MHL (Wills *et al*., 2020). One situational analysis of small island developing states (SIDS) in the region, such as Bermuda, indicated that people in SIDS tend to have low levels of MHL (Walker *et al*., 2022). However, no studies have investigated MHL among adolescents in Bermuda, making it difficult to determine whether mental health promotion should be a policy priority. Despite Bermuda being one of the few high-income countries in the Caribbean (Ministry of Finance Bermuda, 2023), a small population size deprives SIDS like Bermuda of the economies of scale needed to run high-quality health systems (Suzana *et al*., 2018). For example, the health systems of SIDS in the Caribbean are reported to lack a focus on mental health promotion (Walker *et al*., 2022), which may influence adolescents’ ability to recognize mental disorders, their awareness of mental health services and, as a result, who they endorse as helpful for a peer.

The MHL of adolescents in Bermuda may be influenced by demographic variables such as age, gender and race. Yet, there is limited evidence to inform our understanding of how these demographic factors influence MHL among adolescents in the context of Bermuda. Studies that explore these demographic variables are typically conducted with older adolescents attending college in the USA (Kim *et al*., 2015; Cheng *et al*., 2018), or with US or Australian youth samples up to 25 years of age (Cotton *et al*., 2006; Reavley and Jorm, 2011a; Zorrilla *et al*., 2019). Regarding problem recognition, relevant studies indicate problem recognition appears to improve with age (Wright *et al*., 2005; Singh *et al*., 2022), is higher among females compared to males (Cotton *et al*., 2006; Reavley and Jorm, 2011b; Singh *et al*., 2022) and that being White is associated with higher MHL than other racial groups (Benuto *et al*., 2019; Zorrilla *et al*., 2019), particularly with regard to recognizing depression in a vignette (Kim *et al*., 2015). Regarding who adolescents believe are helpful sources of support for a peer with a mental health problem, older age appears to increase the likelihood of suggesting seeking help from psychological services for depression (Swords *et al*., 2011), while males are less likely than females to suggest professional help-seeking (Jorm and Wright, 2007; Olsson and Kennedy, 2010). Studies on how race influences who adolescents believe would be helpful for a peer are not commonly conducted, with more research focused on racial differences in the beliefs about the effectiveness of various forms of treatment such as psychotherapy (Jacobs *et al*., 2008). It is unclear how findings from this type of research may translate to racial differences in beliefs about the helpfulness of sources of support for a peer with a mental health problem.

Earlier findings from this study (D. Cavanagh, submitted for publication) found that adolescents in Bermuda have high levels of depression and anxiety symptoms. The lack of local MHL data limits the ability of policymakers to make informed decisions regarding adolescent mental health promotion (Walker *et al*., 2022). As such, the current study seeks to understand the MHL of adolescents for depression and social anxiety in Bermuda. Specifically, this study investigates how age, gender and race influence disorder recognition and beliefs about the helpfulness of various sources of support for a peer with depression or social anxiety.

## METHODS

### Study population and data collection

This cross-sectional study aimed to survey all adolescent students attending middle and secondary education in Bermuda. According to school records from the 2022–2023 Academic Year the potential participant population was estimated to be 3593 adolescents aged 10–18 years. Students were recruited through all but one of the 16 middle (grades M1–M3) and high (grades S1–S4) schools in Bermuda, which included eight private schools, six public (government-funded) schools and two government-funded alternative education schools. The alternative education schools serve students who are either unable to participate in mainstream education or with severe learning disabilities and complex care needs (who were unable to participate in this study). Historic segregation in schools continues to affect racial differences in enrolment rates between private and public schools as there continues to be overrepresentation of Black students in government-funded schools and underrepresentation of these students in private schools (see *Results* section for further details). A detailed breakdown of the school system in Bermuda, including which schools were surveyed, is provided in (D. Cavanagh, submitted for publication).

Online surveys were administered during students’ regular class time under the supervision of a teacher at their school between 3 November 2022 and 1 June 2023. The survey involved 22 questions including seven matrix-style questions that included a total of 56 items. Pilot testing of the survey showed the median time to complete the questions was ~ 14 min. A backup paper version of the survey was created to cater for any schools that had technical issues on the day of administration. Approval for the study was obtained from the University of Melbourne Human Research Ethics Committee (ID: 23177) and the Bermuda Hospitals Board Institutional Review Board. Further details about ethical considerations and the opt-out consent process are provided in (D. Cavanagh, submitted for publication).

### Measures

The online survey was hosted by the survey platform Qualtrics (*Qualtrics XM—Experience Management Software*, n.d.). It was designed to gather information about students’ demographic characteristics, their MHL and mental health first aid beliefs, their beliefs related to stigma and social distance, their intentions and barriers to help-seeking and their symptoms of depression and anxiety.

The survey presented one of two vignettes depicting a fictional peer of the same age, gender and race as the respondent. The vignettes were adapted from the Australian Youth National Survey of Mental Health Literacy and Stigma (Reavley and Jorm, 2011a). The vignettes presented to participants described symptoms of either major depression or social anxiety disorder as per DSM5 and ICD11 and have been previously used in national studies (Reavley and Jorm, 2011a), including with adolescents (Loureiro *et al*., 2013). Participants were randomized to receive either the depression or the social anxiety vignette, but the character in the vignette was portrayed as the same age, gender and race as the respondent. Participants who identified as female received a vignette labelled as ‘Mikayla’ whereas those who identified as male, or did not identify as male/female, received a vignette labelled as ‘Cameron’. The focus of this article involves the use of the vignettes to examine participants’ ability to correctly recognize depression or social anxiety and their beliefs about sources of support for a peer and how these outcomes are associated with age, gender, race and the presence of symptoms of depression and anxiety. Examples of the vignettes are given in Table 1.

**Table 1:** Vignettes depicting a peer with a mental health problem

Depression	Social anxiety
Cameron^a^ is the same age and race as you. He has been feeling unusually sad and miserable for the last few weeks. He is tired all the time and has trouble sleeping at night. Cameron doesn’t feel like eating and has lost weight. He can’t keep his mind on his studies and his marks have dropped. He puts off making any decisions and even day-to-day tasks seem too much for him. His parents and friends are very concerned about him.	Mikayla^a^ is the same age and race as you. She is living at home with her parents. Since starting her new school last year she has become even more shy than usual and has made only one friend. She would really like to make more friends but is scared that she’ll do or say something embarrassing when she’s around others. Although Mikayla’s work is OK she rarely says a word in class and becomes incredibly nervous, trembles, blushes and seems like she might vomit if she has to answer a question or speak in front of the class. At home, Mikayla is quite talkative with her family, but becomes quiet if anyone she doesn’t know well comes over. She never answers the phone and she refuses to attend social gatherings. She knows her fears are unreasonable but she can’t seem to control them and this really upsets her.

^a^For male participants and for those who selected ‘I identify with another term’, vignettes were presented as ‘Cameron’s story’. For female participants, vignettes were presented as ‘Mikayla’s story’. Participants were randomized to receive either the depression or social anxiety vignettes.

#### Demographic characteristics

First, the survey asked students to indicate the month and year of their birth, and this was later used to calculate their age on the date of survey completion. Next, participants were asked to indicate their gender identity by selecting, in response to the question *Are you…?*: Male, Female or ‘I identify with another term’. The survey then asked *What do you consider yourself to be* and participants indicated their race by selecting one or more of the options: Black, White, Portuguese, Mixed, Asian or Pacific Islander or ‘Other’. The final response option also allowed for open-text entry so that participants could specify what race they identified with.

#### Problem recognition

After being presented with the vignette, respondents were asked *what, if anything, do you think is wrong with [Cameron / Mikayla]?* For the depression vignette, those who wrote ‘depressed’, ‘depression’ or ‘depressive’ were considered to have correctly recognized the disorder and assigned a score of 1.

For the social anxiety vignette, those who wrote ‘social phobia, ‘social anxiety’, ‘anxiety’, ‘anxious’, ‘anxiety attacks’ or ‘anxiety disorder’ were considered to have correctly identified the disorder and assigned a score of 1. Those who incorrectly recognized the disorder were assigned a score of 0. This coding frame was taken from the Teen Mental Health First Aid studies (Hart *et al*., 2022).

#### Beliefs about sources of help for a peer

To assess respondents’ beliefs about various potential sources of help for a peer, respondents were then asked to rate each of the 11 items on *a list of people who Cameron/Mikayla could ask for help* as helpful or harmful to Cameron/Mikayla, or as ‘unsure’. The list of sources of help included: ‘a family doctor’, ‘a counsellor’, ‘Mid-Atlantic Wellness Institute (MAWI) staff member’, ‘a psychologist’, ‘a psychiatrist’ and ‘a school counsellor’ (all coded as ‘clinical professionals’ for analyses) and ‘a close family member’, ‘a close friend’, ‘a teacher’, ‘a coach’ and ‘a priest’ (all coded as ‘lay individuals’ for analyses).

#### Depression and anxiety symptoms

The PHQ-9 (Patient Health Questionnaire-9) and the General Anxiety Disorder-7 (GAD-7), both validated screening tools (Kroenke *et al*., 2001; Spitzer *et al*., 2006), were used to assess symptoms of depression and anxiety, respectively, according to the *Diagnostic and Statistical Manual of Mental Disorders, 5th edition* (American Psychiatric Association, 2013) criteria, over the past 2 weeks. Responses that indicated participants were experiencing moderate to severe symptoms of either depression or anxiety—indicated as having a score of ≥ 10 on either scale—were coded as having these symptoms present and assigned a score of 1, while those who were not experiencing the symptoms were coded as having these symptoms absent and assigned a score of 0. For the PHQ-8, a cut-point score of 10 and above has a sensitivity of 100% and a specificity of 95% for major depressive disorder (Kroenke *et al*., 2009). The GAD-7 sensitivity and specificity using a cut score of 10 are 89% and 82%, respectively (Spitzer *et al*., 2006). The presence or absence of moderate to severe depression/anxiety symptoms was examined as a variable associated with both problem recognition and the beliefs about sources of help for a peer.

### Statistical analyses

Demographic characteristics and correct problem recognition of the disorder in the vignette were first summarized across both vignettes as either mean ± standard deviation for age or *n* (%) for all other variables. For gender, participants who assented and reported ‘I identify with another term’ (*N* = 50) did not fully complete all items in the survey, so further analyses were restricted to male and female categories only. For the purposes of analyses, participants who identified as Mixed (17.4%), Portuguese (6.7%), Asian or Pacific Islander (2.0%) or Other (2.9%) were recoded into a category labelled ‘Minority’.

Next, a chi-square test of independence was used to assess for statistically significant differences in correct problem recognition between the depression and social anxiety vignettes. We then conducted binary logistic regressions to examine whether the independent variables of age, gender, race and the presence of moderate to severe depression symptoms were associated with the ability to correctly recognize the problem in the depression and social anxiety vignettes. With the exception of age, which was a continuous variable, all independent variables were categorical variables. These variables were simultaneously entered into the model, where the variable sub-categories that are italicized were the reference categories for each of the respective variables: gender (*male*, female) and race (*White*, Black, Minority), moderate to severe depression/anxiety symptoms (*absence*, presence).

The data for beliefs about sources of help for a peer were first analysed across vignettes using percent frequencies for the options ‘helpful’, ‘harmful’ or ‘unsure’. Beliefs about each source of help were reported separately under the two categories of clinical professionals and lay individuals. We then conducted binary logistic regressions to examine whether the independent variables of age, gender, race, the presence of moderate to severe depression symptoms and correct problem recognition were associated with the belief that various sources of help would be helpful for a peer. The aforementioned categorical variables were again entered simultaneously with the same reference categories, with the addition of problem recognition where *incorrect problem recognition* was the reference category for correct problem recognition.

Statistical analyses were conducted in SPSS version 29. For regression analyses, only data from participants who assented to the survey and completed all items related to demographic characteristics, problem recognition, beliefs about sources of help for a peer and depression and anxiety symptoms were included in the analysis. Participants who assented but did not complete all items were excluded from the regression analyses for the depression vignette (*n* = 55) and for the social anxiety vignette (*n* = 52). Due to the high number of independent variables, Bonferroni corrections were used in all regression analyses such that only *p*-values < 0.01 were considered statistically significant.

## RESULTS

A total of 2411 adolescents in Bermuda assented to the survey and responded to all the items relating to problem recognition and beliefs about sources of help for a peer. The response rate was 67% and the completion rate among those that started the survey was 89%. On average, these adolescents were aged 13.7 years (SD = ± 2.0 years, range = 10–19 years) and a majority were female (52.8%; male = 47.2%). Of the respondents, 43.7% identified as Black, 27.0% as White and 29.2% as Minority (missing = 0.1%). The majority of participants attended a private school (*n* = 1443), though there were also a large number attending government schools (*n* = 968). Regarding school enrolment, 97.2% and 37.8% of adolescents who identified as White and Black attended private schools, respectively, in comparison to 62.2% of Black adolescents and 2.8% of White adolescents who attended government-funded schools.

Of the participants whose data was included in this study, 50.4% were randomly assigned to either the depression vignette (*N* = 1215) and 49.6% were randomly assigned to the social anxiety vignette (*N* = 1196). For more details about the demographic characteristics, see Table 2.

**Table 2:** Demographic and other characteristics of the sample

Variable	All participants (*N* = 2,411)	Depression vignette (*n* = 1,215)	Social anxiety vignette (*n* = 1,196)
Age, y (± SD)	13.7 ± 2.0	13.7 ± 1.9	13.7 ± 2.0
*Gender, n (%)*			
Female	1,272 (52.8)	638 (52.5)	634 (53.0)
Male	1,139 (47.2)	577 (47.5)	562 (47.0)
*Race, n (%)*			
Black	1,053 (43.7)	546 (44.9)	507 (42.4)
White	651 (27.0)	311 (25.6)	340 (28.4)
Minority^a^	704 (29.2)	357 (29.4)	347 (29.0)
Missing	3 (0.1)	1 (0.1)	3 (0.3)
*School*			
Private	1,443 (59.9)	714 (58.8)	729 (61.0)
Government	968 (40.1)	501 (41.2)	467 (39.0)
*Moderate to severe depression/anxiety symptoms, n (%)*			
Present symptoms	826 (34.3)	413 (34.0)	402 (33.6)
Absent symptoms	1,491 (61.8)	748 (61.6)	743 (62.1)
Missing	105 (4.4)	54 (4.4)	51 (4.3)
*Ability to correctly recognize disorder in vignette, n (%)*			
Correct problem recognition	1,357 (56.3)	723 (59.5)	638 (53.3)

^a^Minority accounted for those who identified as ‘Mixed’ (*N* = 426), ‘Portuguese’ (*N* = 165), ‘Asian or Pacific Islander’ (*N* = 48) or ‘Other’ (*N* = 67).

Participants were randomized to receive either the depression or social anxiety vignette.

Moderate to severe depression/anxiety symptoms indicated by scores of > 10 on the PHQ-8 or GAD-7.

### Problem recognition

More than half of the participants correctly recognized the problem described in the vignette, with 60% correctly recognizing depression (95% CI [56.72, 62.23]) and 53% correctly recognizing social anxiety (95% CI [50.34, 55.99]). For the depression vignette, the most commonly used incorrect label was ‘stress’ (see Supplementary Table S1). For the social anxiety vignette, the most commonly used incorrect label was ‘shy’ (see Supplementary Table S2).

A chi-square test of independence revealed a significant association between the vignette presented (depression or social anxiety) and correct problem recognition *χ*² (1, 2411) = 10.337, *p* < 0.001 with higher correct recognition for depression and a small effect size (Cramer’s *V* = 0.065).

Of the independent variables examined for associations with correctly recognizing depression, one was significant: older age was associated with a greater likelihood of correctly recognizing depression. For the social anxiety vignette, all independent variables except for the presence of moderate to severe depression/anxiety symptoms were significant: older age and female were both associated with a greater likelihood of correctly recognizing social anxiety, while identifying with Black and Minority race were associated with a lower likelihood of correctly recognizing social anxiety. For more detailed results of the odds ratios for these variables, see Table 3.

**Table 3:** Variables associated with adolescents correctly recognizing the problem in the vignette

Variable	Depression vignette (*N* = 1,160)				Social anxiety vignette (*N* = 1,144)			
	OR	99% CI		*p*	OR	99% CI		*p*
Older age (in years)	**1.26**	**1.15**	**1.37**	** < 0.001**	**1.27**	**1.16**	**1.38**	** < 0.001**
Female (reference group: Male)	1.30	0.94	1.80	0.037	**3.45**	**2.46**	**4.85**	** < 0.001**
Black (reference group: White)	1.18	0.80	1.74	0.277	**0.57**	**0.38**	**0.85**	** < 0.001**
Minority (reference group: White)	0.97	0.64	1.48	0.841	**0.61**	**0.39**	**0.95**	**0.004**
Presence moderate to severe depression/anxiety symptoms (reference group: absence)	1.14	0.81	1.61	0.313	1.38	0.96	1.97	0.021

Participants were randomized to receive either the depression or social anxiety vignette.

Moderate to severe depression/anxiety symptoms indicated by scores of  ≥ 10 on the PHQ-8 or GAD-7. Bolded values indicate *p* < 0.01.

### Beliefs about sources of help for a peer

Table 4 shows the percentage frequency of adolescents’ beliefs about various potential sources of help as ‘helpful’, ‘harmful’ or ‘unsure’ for the peer described in the vignette. Across vignettes, the most frequently endorsed sources of help, each with above 80% endorsement as ‘helpful’ for Cameron/Mikayla, were a close family member, a close friend, as well as a counsellor. Across both vignettes, the potential sources of help most commonly rated as ‘harmful’ were a priest/spiritual leader, teacher, coach, as well as a MAWI (a mental health service available to adolescents) staff member. Each of these options was rated as ‘harmful’ by over 10% of adolescents. These four options were also the most frequently rated with ‘unsure’ across both vignettes, with more than one in three adolescents indicating they were ‘unsure’.

**Table 4:** Percentage of adolescents believing sources of help would be helpful, harmful or unsure for a peer with a mental health problem described in a vignette

	Depression vignette (*N* = 1,215)			Social anxiety vignette (*N* = 1,196)		
Variable	Helpful	Harmfull	Unsure	Helpful	Harmful	Unsure
**Clinical professionals**						
A family doctor	74.7	4	21.2	68.1	4.9	27.0
A counsellor	80.9	4.7	14.4	81.8	5.3	13.0
MAWI staff member	52.6	12.2	35.2	42.8	15.2	42.0
A psychologist	66.8	5.5	27.7	62.7	6.8	30.5
A psychiatrist	55.5	7.4	37.1	45.5	9.9	44.6
A school counsellor	68.8	9.7	21.5	71.7	6.4	21.8
**Lay individuals**						
A close family member	82.1	3.5	14.4	87.5	1.8	10.6
A close friend	83.8	3.4	12.8	86.8	2.6	10.6
A teacher	47.5	14.1	38.4	53.3	11.5	35.3
A coach	35.2	12.8	52.0	34.9	12.7	52.4
A priest (or other spiritual/religious leader)	31.9	17.2	50.9	26.4	17.4	56.2

Participants were randomized to receive either the depression or social anxiety vignette.

### Variables associated with beliefs about sources of help for a peer in the vignettes

All independent variables examined (age, gender, Black race, Minority race, correct problem recognition and the presence of moderate to severe depression/anxiety symptoms) were significantly associated with the odds of adolescents believing a potential source of help would be ‘helpful’ for the peer portrayed in the depression or social anxiety vignette. Older age was the most common variable significantly associated with the belief various sources of help would be ‘helpful’. Across vignettes, females, adolescents who identify as White, as well as those who did not correctly identify the problem in the vignette, and those who reported moderate to severe depression/anxiety symptoms, had lower odds of believing various sources of help would be ‘helpful’. The full results are shown in Supplementary Table S3.

## DISCUSSION

Problem recognition is a fundamental component of MHL, as it is considered a necessary first step in seeking help from a professional (Wright *et al*., 2007; Thompson *et al*., 2008), and failure to recognize symptoms can delay help-seeking (Reavley and Jorm, 2011b). In light of these findings, the percentage of adolescents who could correctly recognize depression or social anxiety was less than optimal, especially given there is a high prevalence of depression and anxiety symptoms in adolescents in Bermuda (D. Cavanagh, submitted for publication).

Moreover, while correct rates of recognition of social anxiety in adolescents in Bermuda were higher than in some studies (Olsson and Kennedy, 2010; Reavley and Jorm, 2011b), the rates of depression recognition are lower than in other high-income countries such as Portugal and Australia (Reavley and Jorm, 2011b; Jorm, 2012; Loureiro *et al*., 2013). This is perhaps unsurprising given the lack of mental health promotion typical of SIDS in the Caribbean (Walker *et al*., 2022). In any case, it is concerning that more than two out of five adolescents could not correctly recognize disorders, especially given that we accepted as ‘correct recognition’ responses that used the label depression or anxiety in combination with other normalizing labels such as stress or shy, respectively. The high frequencies of these normalizing labels are problematic, as using them has been found to decrease the likelihood of seeking professional help (Jorm, 2012). The use of these labels over clinical terms may indicate a lack of education for adolescents on the signs and symptoms of these disorders. Alternatively, the use of these labels may reflect the growing global trend of misinformation and self-diagnosis shared on social media platforms to which adolescents are exposed, leading to a distorted understanding of the symptoms of mental illness (Haltigan *et al*., 2023). In either case, our results support calls to improve mental health promotion in SIDS in the Caribbean (Walker *et al*., 2022).

Demographic differences in problem recognition partially supported previous research. Across vignettes, older age was associated with greater correct problem recognition, while female gender was associated with greater correct problem recognition for social anxiety. These results are in line with previous studies that show MHL improves with age and is higher in females compared to males (Wright *et al*., 2005; Burns and Rapee, 2006; Cotton *et al*., 2006; Kaneko and Motohashi, 2007; Singh *et al*., 2022). Yet, it was surprising that there was no significant gender difference for recognition in the depression vignette and this result is unlikely due to a ceiling effect for depression recognition found in other studies (Tan *et al*., 2021; Wilcox *et al*., 2023). In terms of racial differences, adolescents who identified as White were more likely to correctly recognize the problem in the social anxiety vignette than those who identified as Black or Minority. One possible explanation may be due to the historic racial segregation of schools in Bermuda, which still lingers today as Black adolescents are overrepresented in government-funded schools and underrepresented in private schools. Studies in the USA have shown that social anxiety is more common in White adolescents compared to Black and Minority adolescents (Compton *et al*., 2000; Asnaani *et al*., 2010), meaning White adolescents have more exposure to this disorder through their peers at school and as a result may be better at recognizing the symptoms. However, the racial differences in correctly recognizing social anxiety disappeared when narrowing the generous coding frame, which included any mention of ‘anxiety’, to only correctly code responses that specifically referred to ‘social anxiety’ (see Supplementary Table S4). Moreover, there was no difference in correct problem recognition between races for the depression vignette, a result which contradicts research from the USA indicating that White young adults are more likely to correctly recognize depression in a vignette than their Black counterparts (Kim *et al*., 2015). Further research would be useful in understanding the driving factors of racial differences in problem recognition for social anxiety among adolescents in Bermuda.

Results regarding beliefs about sources of help for a peer are encouraging and have implications for policymakers. The finding that a close friend had the most positive beliefs of helpfulness from adolescents in Bermuda suggests a need for the implementation of programs that aim to improve adolescent MHL, such as the Teen Mental Health First Aid Program which has been found to improve problem recognition (Hart *et al*., 2016, 2022). Regarding professional help-seeking, over half of adolescents endorsed as helpful for a peer all clinical professionals for depression and over 40% endorsed all clinical professionals for a peer with social anxiety. Across both vignettes, the particularly high endorsement was found for a counsellor, at levels similar to a close family member or friend, while three out of four adolescents endorsed a family doctor for a peer with depression. The high levels of endorsement for family doctors suggest training primary care professionals serving adolescents in identifying mental illness symptoms may act as effective early intervention. Many programs exist that focus on adults working with young people, such as the Youth Mental Health First Aid program, which has been found to improve problem recognition (Ng *et al*., 2021). In any case, these findings are promising as positive community attitudes towards clinical professionals increase the likelihood that adolescents will receive appropriate care. Yet, the high rates of endorsement do not extend to all clinical professionals and lay individuals. The issue of stigma (Roberts *et al*., 2013) and the challenges associated with maintaining confidentiality of mental health problems, specifically in small island communities like Bermuda (Malik and Simmons, 2012; Walker *et al*., 2022), may be negatively affecting beliefs about sources of help being helpful. Creative and technological solutions may be required to further improve community attitudes towards mental healthcare.

Gender and racial differences in who was endorsed as helpful were surprising. For depression, compared to females, males endorsed more sources including a teacher, a coach, a priest and, most surprisingly, a family doctor. This finding contradicts previous research indicating that male adolescents were less likely to recommend professional help-seeking (Jorm and Wright, 2007; Olsson and Kennedy, 2010). A partial explanation may be that unlike previous research (Wright *et al*., 2005; Burns and Rapee, 2006; Cotton *et al*., 2006; Kaneko and Motohashi, 2007), there were no gender differences in depression recognition. Further research would be useful in understanding why females report more negative community attitudes towards sources of help. Similarly, further research would be useful in understanding the novel finding that there were more sources of help endorsed among Black and Minority adolescents compared to White adolescents. In particular, it would be interesting to see if their greater endorsement of support for a peer correlated with beliefs about these sources of help for themselves and their own help-seeking intentions.

The most concerning finding was that across vignettes the presence of moderate to severe depression/anxiety symptoms was associated with a lower likelihood of believing that a family doctor, a counsellor, a school counsellor and a family member were helpful. One possible explanation comes from the finding that experiencing depression symptoms reduces the likelihood that adolescents seek help (Wilson *et al*., 2007), which may in turn influence adolescents’ belief that sources of help would be helpful for their peers. Indeed, supplementary analyses describing the relationship between increasing PHQ scores and the percentage who endorsed a family doctor, a counsellor and a school counsellor as helpful showed that the more severe PHQ-8 symptoms reported the less likely these sources of help were considered helpful for a peer (see Supplementary Table S5). This explanation gives greater support for the implementation of programs seeking to improve MHL to challenge those beliefs. Alternatively, it may suggest a need to improve either or both the actual and perceived quality of care delivered to adolescents.

### Strengths and limitations

This study is the first to investigate the MHL of adolescents in Bermuda. Investigating depression and social anxiety literacy can lead to a deeper understanding of adolescents’ ability to recognize disorders common mental disorders, and who they endorse as sources of help for a peer. In addition, among studies investigating how demographic variables influence problem recognition, most are focused on depression and other disorders such as schizophrenia (Cotton *et al*., 2006) or general anxiety disorder (Kim *et al*., 2015; Cheng *et al*., 2018), with few studies investigating social anxiety (Reavley and Jorm, 2011a). This study is limited by the inability to control for a number of confounding variables. For example, the language skill of participants may influence the ability to understand and label the vignettes. This study is also limited in that we examined beliefs about sources of help in relation to a person described in a vignette rather than actual recommendations provided to a peer or personal help-seeking intentions. Moreover, while previous studies in Australia have shown that correct problem recognition is associated with improved help-seeking intentions (Wright *et al*., 2007; Thompson *et al*., 2008), a more recent study has found that correct problem recognition was not associated with the intention to seek help among adolescents in Bermuda (D. Cavanagh, submitted for publication). As such, further research is needed to understand what factors other than MHL are influencing help-seeking among adolescents in Bermuda. Finally, the use of vignettes has been criticized for lacking ecological validity in that in real-life situations individuals likely get a good deal more information about symptoms in others, particularly in close relationships (Furnham and Swami, 2018). Therefore, what adolescents suggest to their peers as helpful for their mental health problem is highly complex and depends on multiple contextual factors.

## CONCLUSION

Recognition rates of depression and social anxiety among adolescents in Bermuda were sub-optimal. Similarly, while counsellors had a high endorsement from adolescents, many clinical and lay professionals were not endorsed, with a small minority considering these sources of help-seeking to actually be harmful. Our results strongly support the introduction of programs that aim to improve problem recognition the peer-to-peer Teen Mental Health First Aid Program or the adult Youth Mental Health First Aid program recognition (Ng *et al*., 2021). The latter program has recently been introduced on the island and can be scaled up and offered to parents, teachers, coaches and priests, all of whom can play an important role in helping adolescents developing a mental health problem or experiencing a mental health crisis. Further research to understand surprising gender and racial differences in MHL, potential issues of confidentiality, high levels of stigma and perceived or actual poor quality of care would be useful in tailoring programs aimed at improving MHL and help-seeking.

## Supplementary Material

daae131_suppl_Supplementary_Material

## Data Availability

Access to the data described within the manuscript and supplementary files can be granted upon an email request to the authors.
