# Supplementary material for: Mental health literacy of adolescents in Bermuda, according to age, gender and race
Source: Health Promot Int. 2024 Oct 14;39(5):daae131. doi: 10.1093/heapro/daae131 (PMC11471998; doi:10.1093/heapro/daae131)
Supplement: daae131_suppl_Supplementary_Material [file daae131_suppl_supplementary_material.docx]

| Supplementary Table 1 Number and frequency of respondents mentioning each label to describe the problem shown in the depression vignette (n = 1215) | | |
| --- | --- | --- |
| **Labels** | **N** | % |
| Depression, depressive, depressed* | 723 | 59.5 |
| Suicidal | 2 | 0.2 |
| Anxiety | 21 | 1.7 |
| Eating disorder | 20 | 1.6 |
| Mental health problem | 33 | 2.7 |
| Sleep problems | 33 | 2.7 |
| Stress | 88 | 7.2 |
| Eating issues | 15 | 1.2 |
| Sad | 38 | 3.1 |
| Self conscious / self esteem / confidence issues | 12 | 1 |
| Problems at school | 10 | 0.8 |
| Bullying | 46 | 3.8 |
| Significant life event | 7 | 0.6 |
| Interpersonal problem | 24 | 2 |
| Physical health problem | 2 | 0.2 |
| Other specified mental health problem | 5 | 0.4 |
| Other | 76 | 6.2 |
| Stigmatising response | 7 | 0.6 |
| Nothing wrong | 3 | 0.2 |
| Not sure | 12 | 1 |
| Uncodeable | 38 | 3.1 |
| * These responses considered ‘correct’ for the depression vignette |  |  |
| ** These responses included descriptions of the peer in the vignette such as ‘lazy’ or ‘lacking discipline’ |  |  |

| Supplementary Table 2 Number and frequency of respondents mentioning each label to describe the problem shown in the social anxiety vignette (n = 1196) | | |
| --- | --- | --- |
| **Labels** | **N** | % |
| Social anxiety / social phobia* | 441 | 36.9 |
| Anxiety / anxiety attacks / anxious* | 193 | 16.1 |
| Anxiety disorder (unspecified)* | 2 | 0.2 |
| Depression | 11 | 0.9 |
| Shy | 148 | 12.4 |
| Self conscious / self esteem / confidence issues | 34 | 2.8 |
| Mental health problem | 2 | 0.2 |
| Scared | 60 | 5 |
| Nervous / uncomfortable | 38 | 3.2 |
| Introverted | 23 | 1.9 |
| Socially awkward | 5 | 0.4 |
| Difficuty socialising | 23 | 1.9 |
| Anti-social | 36 | 3 |
| Autism | 1 | 0.1 |
| Bullying | 4 | 0.3 |
| Significant life event | 2 | 0.2 |
| Other specified mental health problem | 3 | 0.2 |
| Other | 49 | 4.1 |
| Stigmatising response | 6 | 0.5 |
| Nothing wrong | 54 | 4.5 |
| Not sure | 17 | 1.4 |
| Uncodeable | 45 | 3.7 |
| * These responses considered ‘correct’ for the social anxiety vignette |  |  |
| ** These responses included descriptions of the peer in the vignette such as ‘not brave or ‘unlikeable’ |  |  |

| Supplementary Table 3 Summary of logistic regressions for variables associated with sources of help for a peer described in a vignette that adolescents would consider 'helpful' | | | | | | | | | |  |
| --- | --- | --- | --- | --- | --- | --- | --- | --- | --- | --- |
| Variable | Depression vignette (N = 1160) | | | |  | Social anxiety vignette (N = 1144) | | | |  |
|  | OR | 99% CI |  | p |  | OR | 99% CI |  | p |  |
|  | *A family doctor* | | | | |  |  |  |  |  |
| Older age (in years) | 0.93 | 0.84 | 1.02 | 0.034 |  | **0.90** | **0.83** | **0.98** | **0.002** |  |
| Female (reference group: Male) | **0.53** | **0.36** | **0.77** | **<.001** |  | 0.89 | 0.62 | 1.26 | 0.375 |  |
| Black (reference group: White) | 0.99 | 0.64 | 1.54 | 0.968 |  | 1.11 | 0.74 | 1.66 | 0.501 |  |
| Minority (reference group: White) | 0.90 | 0.56 | 1.46 | 0.586 |  | 1.12 | 0.72 | 1.74 | 0.503 |  |
| Correct problem recognition (reference group: incorrect) | 1.28 | 0.89 | 1.86 | 0.084 |  | 1.08 | 0.76 | 1.55 | 0.559 |  |
| Presence moderate to severe depression/anxiety symptoms (reference group: absence) | **0.64** | **0.44** | **0.93** | **0.002** |  | **0.58** | **0.41** | **0.83** | **<.001** |  |
|  | *A counselor* | | | |  |  |  |  |  |  |
| Older age (in years) | 0.98 | 0.88 | 1.08 | 0.566 |  | 1.02 | 0.92 | 1.13 | 0.681 |  |
| Female (reference group: Male) | 1.22 | 0.82 | 1.82 | 0.203 |  | 1.48 | 0.96 | 2.27 | 0.019 |  |
| Black (reference group: White) | **1.66** | **1.04** | **2.67** | **0.006** |  | **1.86** | **1.15** | **3.00** | **<.001** |  |
| Minority (reference group: White) | 1.18 | 0.72 | 1.94 | 0.383 |  | **1.70** | **1.01** | **2.84** | **0.008** |  |
| Correct problem recognition (reference group: incorrect) | **1.56** | **1.05** | **2.33** | **0.004** |  | 1.26 | 0.82 | 1.95 | 0.167 |  |
| Presence moderate to severe depression/anxiety symptoms (reference group: absence) | **0.62** | **0.41** | **0.94** | **0.003** |  | **0.56** | **0.36** | **0.85** | **<.001** |  |
|  | *A Mid-Atlantic Wellness Institute (MAWI) staff member* | | | | | |  |  |  |  |
| Older age (in years) | 1.01 | 0.93 | 1.10 | 0.728 |  | 0.94 | 0.87 | 1.02 | 0.055 |  |
| Female (reference group: Male) | 0.87 | 0.64 | 1.19 | 0.26 |  | 0.75 | 0.54 | 1.06 | 0.03 |  |
| Black (reference group: White) | 0.76 | 0.52 | 1.11 | 0.061 |  | 0.74 | 0.50 | 1.08 | 0.037 |  |
| Minority (reference group: White) | 0.86 | 0.57 | 1.30 | 0.348 |  | 0.71 | 0.47 | 1.08 | 0.035 |  |
| Correct problem recognition (reference group: incorrect) | 1.31 | 0.95 | 1.80 | 0.031 |  | **1.80** | **1.28** | **2.54** | **<.001** |  |
| Presence moderate to severe depression/anxiety symptoms (reference group: absence) | 0.84 | 0.61 | 1.17 | 0.175 |  | 0.77 | 0.54 | 1.08 | 0.045 |  |
|  | *A psychologist* | | | | |  |  |  |  |  |
| Older age (in years) | **1.15** | **1.05** | **1.25** | **<.001** |  | **1.17** | **1.08** | **1.28** | **<.001** |  |
| Female (reference group: Male) | 0.87 | 0.62 | 1.22 | 0.291 |  | 0.91 | 0.64 | 1.29 | 0.499 |  |
| Black (reference group: White) | 0.86 | 0.57 | 1.29 | 0.342 |  | 0.95 | 0.64 | 1.41 | 0.738 |  |
| Minority (reference group: White) | 0.82 | 0.53 | 1.27 | 0.24 |  | 0.97 | 0.63 | 1.49 | 0.851 |  |
| Correct problem recognition (reference group: incorrect) | 1.40 | 1.00 | 1.96 | 0.01 |  | **1.85** | **1.30** | **2.61** | **<.001** |  |
| Presence moderate to severe depression/anxiety symptoms (reference group: absence) | 1.16 | 0.81 | 1.65 | 0.292 |  | 1.06 | 0.74 | 1.51 | 0.695 |  |
|  | *A psychiatrist* | |  |  |  |  |  |  |  |  |
| Older age (in years) | **1.17** | **1.08** | **1.27** | **<.001** |  | **1.09** | **1.01** | **1.18** | **0.005** |  |
| Female (reference group: Male) | 0.87 | 0.63 | 1.20 | 0.265 |  | 1.00 | 0.72 | 1.40 | 0.998 |  |
| Black (reference group: White) | 0.71 | 0.48 | 1.04 | 0.021 |  | 0.95 | 0.65 | 1.39 | 0.714 |  |
| Minority (reference group: White) | 0.82 | 0.54 | 1.25 | 0.232 |  | 1.10 | 0.73 | 1.66 | 0.543 |  |
| Correct problem recognition (reference group: incorrect) | 1.37 | 0.99 | 1.89 | 0.012 |  | **1.74** | **1.25** | **2.44** | **<.001** |  |
| Presence moderate to severe depression/anxiety symptoms (reference group: absence) | 1.22 | 0.87 | 1.71 | 0.127 |  | 1.07 | 0.76 | 1.50 | 0.612 |  |
|  | *A school counselor* | | | | |  |  |  |  |  |
| Older age (in years) | 0.97 | 0.88 | 1.05 | 0.297 |  | 0.94 | 0.86 | 1.03 | 0.069 |  |
| Female (reference group: Male) | 0.95 | 0.67 | 1.34 | 0.686 |  | 1.28 | 0.89 | 1.85 | 0.085 |  |
| Black (reference group: White) | **1.55** | **1.03** | **2.33** | **0.006** |  | 1.29 | 0.85 | 1.95 | 0.122 |  |
| Minority (reference group: White) | 1.14 | 0.74 | 1.76 | 0.434 |  | 1.22 | 0.77 | 1.91 | 0.266 |  |
| Correct problem recognition (reference group: incorrect) | 1.24 | 0.88 | 1.76 | 0.11 |  | 0.86 | 0.59 | 1.25 | 0.307 |  |
| Presence moderate to severe depression/anxiety symptoms (reference group: absence) | **0.49** | **0.34** | **0.69** | **<.001** |  | **0.55** | **0.38** | **0.79** | **<.001** |  |
|  | *A close family member* | | | |  |  |  |  |  |  |
| Older age (in years) | **0.89** | **0.80** | **0.99** | **0.004** |  | 0.91 | 0.81 | 1.02 | 0.031 |  |
| Female (reference group: Male) | 0.74 | 0.48 | 1.13 | 0.065 |  | 0.85 | 0.51 | 1.40 | 0.392 |  |
| Black (reference group: White) | 1.05 | 0.65 | 1.72 | 0.786 |  | 1.24 | 0.70 | 2.18 | 0.328 |  |
| Minority (reference group: White) | 1.20 | 0.69 | 2.06 | 0.396 |  | 1.21 | 0.66 | 2.23 | 0.424 |  |
| Correct problem recognition (reference group: incorrect) | 0.92 | 0.60 | 1.41 | 0.615 |  | 1.05 | 0.63 | 1.74 | 0.822 |  |
| Presence moderate to severe depression/anxiety symptoms (reference group: absence) | **0.46** | **0.31** | **0.70** | **<.001** |  | **0.56** | **0.34** | **0.91** | **0.002** |  |
|  | *A close friend* | | | | |  |  |  |  |  |
| Older age (in years) | **1.14** | **1.01** | **1.27** | **0.004** |  | **1.16** | **1.03** | **1.31** | **0.002** |  |
| Female (reference group: Male) | 0.96 | 0.63 | 1.48 | 0.813 |  | 1.06 | 0.65 | 1.71 | 0.77 |  |
| Black (reference group: White) | 0.86 | 0.51 | 1.43 | 0.436 |  | 0.76 | 0.44 | 1.32 | 0.208 |  |
| Minority (reference group: White) | 1.07 | 0.60 | 1.90 | 0.77 |  | 1.09 | 0.58 | 2.04 | 0.728 |  |
| Correct problem recognition (reference group: incorrect) | 0.97 | 0.63 | 1.50 | 0.851 |  | 1.25 | 0.77 | 2.02 | 0.242 |  |
| Presence moderate to severe depression/anxiety symptoms (reference group: absence) | 0.78 | 0.50 | 1.21 | 0.141 |  | 0.95 | 0.58 | 1.55 | 0.778 |  |
|  | *A teacher* | | | |  |  |  |  |  |  |
| Older age (in years) | 1.02 | 0.94 | 1.11 | 0.53 |  | 1.01 | 0.93 | 1.09 | 0.739 |  |
| Female (reference group: Male) | **0.71** | **0.52** | **0.97** | **0.005** |  | 0.99 | 0.71 | 1.38 | 0.948 |  |
| Black (reference group: White) | 1.45 | 0.99 | 2.11 | 0.012 |  | 0.98 | 0.67 | 1.42 | 0.881 |  |
| Minority (reference group: White) | 1.20 | 0.80 | 1.82 | 0.253 |  | 1.08 | 0.72 | 1.63 | 0.612 |  |
| Correct problem recognition (reference group: incorrect) | 0.92 | 0.66 | 1.26 | 0.476 |  | 0.78 | 0.56 | 1.09 | 0.059 |  |
| Presence moderate to severe depression/anxiety symptoms (reference group: absence) | **0.65** | **0.47** | **0.91** | **<.001** |  | 0.77 | 0.55 | 1.08 | 0.045 |  |
| *A coach* | |  |  |  |  |  |  |  |  |  |
| Older age (in years) | **1.24** | **1.14** | **1.36** | **<.001** |  | **1.20** | **1.10** | **1.30** | **<.001** |  |
| Female (reference group: Male) | **0.51** | **0.36** | **0.71** | **<.001** |  | **0.68** | **0.48** | **0.97** | **0.005** |  |
| Black (reference group: White) | 1.29 | 0.86 | 1.94 | 0.108 |  | 1.01 | 0.67 | 1.50 | 0.975 |  |
| Minority (reference group: White) | 1.27 | 0.81 | 1.98 | 0.17 |  | 1.08 | 0.70 | 1.66 | 0.646 |  |
| Correct problem recognition (reference group: incorrect) | 0.78 | 0.55 | 1.09 | 0.055 |  | 0.85 | 0.60 | 1.21 | 0.238 |  |
| Presence moderate to severe depression/anxiety symptoms (reference group: absence) | 0.97 | 0.68 | 1.38 | 0.806 |  | 0.97 | 0.68 | 1.38 | 0.798 |  |
| *A priest* | |  |  |  |  |  |  |  |  |  |
| Older age (in years) | 1.03 | 0.94 | 1.13 | 0.382 |  | 1.02 | 0.93 | 1.12 | 0.557 |  |
| Female (reference group: Male) | **0.63** | **0.45** | **0.90** | **<.001** |  | 0.86 | 0.59 | 1.25 | 0.306 |  |
| Black (reference group: White) | **3.76** | **2.35** | **6.03** | **<.001** |  | **3.11** | **1.92** | **5.02** | **<.001** |  |
| Minority (reference group: White) | **2.53** | **1.52** | **4.20** | **<.001** |  | **2.37** | **1.41** | **3.97** | **<.001** |  |
| Correct problem recognition (reference group: incorrect) | 0.91 | 0.64 | 1.29 | 0.476 |  | 0.86 | 0.59 | 1.26 | 0.313 |  |
| Presence moderate to severe depression/anxiety symptoms (reference group: absence) | 0.98 | 0.68 | 1.41 | 0.88 |  | 0.83 | 0.56 | 1.21 | 0.196 |  |
| Participants were randomized to receive either the depression or social anxiety vignette | | | | | | | |  |  |  |
| Moderate to severe depression/anxiety symptoms indicated by scores of ≥ 10 on the PHQ-8 or GAD-7 | | | | | | | | | | |

| Supplementary Table 4 Variables associated with adolescents correctly using the term 'social anxiety' to identify the problem in the social anxiety vignette | | | | | |
| --- | --- | --- | --- | --- | --- |
| Variable |  | Social anxiety vignette (N = 1144) | | | |
|  |  | OR | 99% CI |  | p |
| Older age (in years) |  | **1.24** | **1.14** | **1.34** | **<.001** |
| Female (reference group: Male) |  | **2.81** | **1.98** | **3.99** | **<.001** |
| Black (reference group: White) |  | 0.74 | 0.49 | 1.10 | 0.052 |
| Minority (reference group: White) |  | 0.79 | 0.51 | 1.23 | 0.171 |
| Presence of moderate to severe depression or anxiety symptoms (reference group: absence) |  | 1.33 | 0.93 | 1.89 | 0.039 |
| Participants were randomised to receive either the depression or social anxiety vignette | | | | | |
| Moderate to severe depression/anxiety symptoms indicated by scores of ≥10 on the PHQ-8 or GAD-7 | | | | | |

| Supplementary Table 5 Summary of logistic regressions for variables associated with sources of help for a peer described in a vignette that adolescents would consider 'helpful' | | | | | |
| --- | --- | --- | --- | --- | --- |
| Variable | Depression vignette (N = 1186) | | | | |
|  | OR | | 99% CI |  | p |
|  | *A family doctor* | | | | |
| Older age (in years) | 0.92 | | 0.84 | 1.01 | 0.028 |
| Female (reference group: Male) | **0.58** | | **0.40** | **0.85** | **<.001** |
| Black (reference group: White) | 0.99 | | 0.64 | 1.54 | 0.964 |
| Minority (reference group: White) | 0.96 | | 0.59 | 1.55 | 0.826 |
| Correct problem recognition (reference group: incorrect) | 1.32 | | 0.91 | 1.91 | 0.057 |
| Mild depressive symptoms (reference group: no symptoms) | **0.56** | | **0.36** | **0.86** | **<.001** |
| Moderate depressive symptoms (reference group: no symptoms) | 0.61 | | 0.36 | 1.03 | 0.014 |
| Moderately severe to severe depression symptoms (reference group: no symptoms) | **0.29** | | **0.16** | **0.51** | **<.001** |
|  | *A counselor* | | | | |
| Older age (in years) | 0.97 | | 0.88 | 1.07 | 0.447 |
| Female (reference group: Male) | 1.25 | | 0.84 | 1.88 | 0.151 |
| Black (reference group: White) | **1.61** | | **1.01** | **2.57** | **0.009** |
| Minority (reference group: White) | 1.20 | | 0.73 | 1.97 | 0.338 |
| Correct problem recognition (reference group: incorrect) | **1.56** | | **1.05** | **2.32** | **0.004** |
| Mild depressive symptoms (reference group: no symptoms) | 0.80 | | 0.50 | 1.28 | 0.224 |
| Moderate depressive symptoms (reference group: no symptoms) | 1.13 | | 0.61 | 2.08 | 0.607 |
| Moderately severe to severe depression symptoms (reference group: no symptoms) | **0.40** | | **0.22** | **0.75** | **<.001** |
|  | *A school counselor* | | | | |
| Older age (in years) | 0.95 | | 0.87 | 1.04 | 0.139 |
| Female (reference group: Male) | 0.93 | | 0.66 | 1.31 | 0.579 |
| Black (reference group: White) | 1.47 | | 0.98 | 2.19 | 0.014 |
| Minority (reference group: White) | 1.14 | | 0.74 | 1.75 | 0.438 |
| Correct problem recognition (reference group: incorrect) | 1.23 | | 0.88 | 1.74 | 0.114 |
| Mild depressive symptoms (reference group: no symptoms) | 0.71 | | 0.48 | 1.07 | 0.031 |
| Moderate depressive symptoms (reference group: no symptoms) | **0.59** | | **0.37** | **0.95** | **0.004** |
| Moderately severe to severe depression symptoms (reference group: no symptoms) | **0.41** | | **0.23** | **0.71** | **<.001** |
|  | *A close family member* | | | | |
| Older age (in years) | **0.88** | | **0.80** | **0.98** | **0.002** |
| Female (reference group: Male) | 0.69 | | 0.45 | 1.04 | 0.021 |
| Black (reference group: White) | 0.99 | | 0.61 | 1.61 | 0.965 |
| Minority (reference group: White) | 1.14 | | 0.66 | 1.96 | 0.532 |
| Correct problem recognition (reference group: incorrect) | 0.93 | | 0.61 | 1.41 | 0.639 |
| Mild depressive symptoms (reference group: no symptoms) | 0.71 | | 0.43 | 1.17 | 0.079 |
| Moderate depressive symptoms (reference group: no symptoms) | **0.41** | | **0.24** | **0.70** | **<.001** |
| Moderately severe to severe depression symptoms (reference group: no symptoms) | 0.59 | | 0.30 | 1.14 | 0.039 |
| Participants were randomised to receive either the depression or social anxiety vignette | |  |  |  |  |
| Mild depression symptoms indicated by scores of 5-9 on the PHQ-8 |  | |  |  |  |
| Moderate depression symptoms indicated by scores of 10-14 on the PHQ-8 |  | |  |  |  |
| Moderately severe depression symptoms indicated by scores of 15+ on the PHQ-8 |  | |  |  |  |
